# Possible Involvement of Leptin in Pathogenesis of Periodontal Disease

**DOI:** 10.3390/biology14101454

**Published:** 2025-10-20

**Authors:** Małgorzata Kozak, Agata Poniewierska-Baran, Michał Czerewaty, Karolina Łuczkowska, Małgorzata Mazurek-Mochol, Bogusław Machaliński, Andrzej Pawlik

**Affiliations:** 1 Department of Dental Prosthetics, Pomeranian Medical University, 70-111 Szczecin, Poland; gosia-ko@o2.pl; 2 Institute of Biology, University of Szczecin, 71-412 Szczecin, Poland; agata.poniewierska-baran@usz.edu.pl; 3 Department of Physiology, Pomeranian Medical University, 70-111 Szczecin, Poland; michal.czerewaty@pum.edu.pl; 4 Department of General Pathology, Pomeranian Medical University, 70-111 Szczecin, Poland; karolina.luczkowska@pum.edu.pl (K.Ł.); machalin@pum.edu.pl (B.M.); 5 Department of Periodontology, Pomeranian Medical University, 70-111 Szczecin, Poland; malgorzata.mazurek@poczta.onet.pl

**Keywords:** leptin, periodontitis, stimulation

## Abstract

**Simple Summary:**

Periodontitis involves chronic inflammation of the periodontal tissues, primarily caused by a bacterial infection that triggers an immune system response. Recent studies have highlighted the effect of leptin on periodontal tissues, particularly on fibroblasts of the periodontal ligament. Periodontal ligament fibroblasts form the main cell population within the ligament and are responsible for regulating tissue homeostasis as well as activating the immune system. They secrete numerous pro-inflammatory mediators, cytokines, and chemokines involved in the development of inflammation in periodontal tissues. The periodontal ligament itself can be a source of many pro-inflammatory mediators. The aim of this study was to examine the effect of leptin on periodontal ligament cells and their secretion of selected pro-inflammatory mediators that may contribute to the pathogenesis of periodontal disease. The results of this study suggest that leptin may contribute to the pathogenesis of periodontitis by modulating the expression of certain pro-inflammatory cytokines in periodontal ligament cells.

**Abstract:**

Periodontitis is a chronic inflammatory condition of the periodontal tissues, ultimately leading to their destruction. The periodontal ligament is a key structure that not only secures the teeth within the alveolus but can also act as a source of numerous mediators involved in the development of inflammation in periodontal tissues. The aim of this study was to investigate the effect of leptin on periodontal ligament cells and their secretion of selected pro-inflammatory mediators that may contribute to the pathogenesis of periodontal disease. The study was conducted on cultured periodontal ligament cells stimulated with leptin. The effect of leptin was assessed on the expression of selected cytokines implicated in the pathogenesis of periodontal disease (IL-1, IL-6, IL-8, IL-10, IL-17, IL-18, and tumour necrosis factor-alpha [TNF-α]) at the mRNA level, as well as on the protein concentrations of these cytokines in culture supernatants. Assessments were carried out after 12, 24, and 48 h of leptin stimulation. The results showed a statistically significant effect of leptin on IL-6 and IL-8 expression at both the mRNA and protein levels. For IL-1, a transient increase in mRNA expression and protein concentration was observed, persisting up to 24 h. A decrease in IL-10 mRNA expression was noted after 48 h of leptin stimulation, with no corresponding effect on IL-10 protein concentration. No significant effect of leptin was found on IL-17 or IL-18 protein concentrations in periodontal ligament cell cultures. These findings suggest that leptin may contribute to the pathogenesis of periodontitis by modulating the expression of certain pro-inflammatory cytokines in periodontal ligament cells.

## 1. Introduction

Periodontitis is most often caused by a bacterial infection in the periodontal tissues, which triggers an immune response [1]. The immune response aimed at eradicating such an infection can often lead to the activation of immune pathways involving various cells that secrete numerous pro-inflammatory mediators, including cytokines, chemokines, and adipokines [2]. There are complex interrelationships between individual immune cells and the secreted pro-inflammatory mediators, which can mutually enhance or inhibit their secretion. This, in turn, increases inflammation within the periodontal tissues and contributes to the destruction of the alveolar process. These processes are driven by numerous pro-inflammatory mediators, which further exacerbate them [3].

An important structure in this context is the periodontal ligament, which serves both as a crucial means of anchoring the teeth within the alveolus and as a potential source of mediators involved in the development of periodontal inflammation [4]. Studies in recent years have shown that alongside numerous cytokines and chemokines, hormones secreted by adipose tissue—known as adipokines—are also implicated in the pathogenesis of periodontal disease [5]. Adipokines participate in various signalling pathways involving cytokines and other pro-inflammatory mediators.

Population studies have demonstrated a higher prevalence of periodontal disease in obese individuals, suggesting a possible role for adipokines in the development of periodontitis [6]. One key adipokine associated with obesity and pro-inflammatory activity is leptin. Produced in adipose tissue, leptin plays a role in regulating metabolism [7,8] but has also been shown to influence immune processes and inflammation. It promotes the proliferation and survival of immune cells and enhances the secretion of numerous pro-inflammatory mediators [9]. Furthermore, leptin has been shown to affect the migration and chemotaxis of many cells involved in inflammatory processes, including monocytes, macrophages, neutrophils, eosinophils, basophils, natural killer cells, and dendritic cells [10]. Thus, leptin appears to induce and amplify inflammation by acting on a range of immune cells. Elevated plasma leptin levels in obese individuals have been linked to low-grade inflammation and an increased risk of cardiovascular disease, type II diabetes, and autoimmune conditions [11].

Recent studies have highlighted the effect of leptin on periodontal tissues, particularly on fibroblasts of the periodontal ligament [12,13]. Periodontal ligament fibroblasts form the main cell population within the ligament and are responsible for regulating tissue homeostasis as well as activating the immune system. They secrete numerous pro-inflammatory mediators, cytokines, and chemokines involved in the development of inflammation in periodontal tissues [13]. In addition, periodontal ligament fibroblasts produce several mediators associated with bone resorption processes, such as receptor activator of nuclear factor-κB (RANK), receptor activator of nuclear factor-κB ligand (RANKL), and nuclear factor-κB. RANKL binds to RANK, enabling the differentiation of progenitor cells into osteoclasts, ultimately leading to alveolar bone destruction [12].

Previous research has indicated a link between leptin and the development of periodontal disease, although findings remain inconclusive. Some studies have reported increased leptin levels in patients with periodontitis, while others have shown decreased levels [14,15,16,17,18]. It has also been observed that the presence of periodontitis may raise plasma leptin levels. Furthermore, plasma leptin concentrations in periodontitis patients have been shown to correlate with pro-inflammatory cytokines implicated in disease pathogenesis, such as interleukin (IL)-1, IL-6, and tumour necrosis factor-alpha (TNF-α) [16].

Leptin influences immune responses and inflammation, both of which contribute to the development of periodontitis and are closely interrelated processes [19,20]. As a defence mechanism against infection, the immune system is activated to combat pathogens. However, excessive stimulation of this response can lead to the production of inflammatory mediators that, while targeting the pathogen, may also cause inflammation within the periodontal tissues and result in tissue damage [21]. Activation of immune cells by pro-inflammatory factors intensifies their chemotaxis to sites of inflammation. These cells release numerous cytotoxic and pro-inflammatory mediators which, although effective against pathogens, can simultaneously damage periodontal tissues [22]. The periodontal ligament itself can be a source of many pro-inflammatory mediators. The aim of this study was to examine the effect of leptin on periodontal ligament cells and their secretion of selected pro-inflammatory mediators that may contribute to the pathogenesis of periodontal disease.

## 2. Materials and Methods

### 2.1. Obtaining Human Periodontal Ligament Cells for Cell Cultures

Periodontal ligament tissue was obtained from teeth extracted from healthy individuals for orthodontic reasons. Isolation and culture of human periodontal ligament cells in vitro were carried out according to a previously described procedure [23]. The tissues were minced and digested in a solution containing collagenase I (1 mg/mL) and collagenase II (0.5 mg/mL) (Sigma-Aldrich, St. Louis, MO, USA) for 120 min at 37 °C, then filtered through a 70-µm cell strainer and centrifuged in phosphate-buffered saline for 8 min at 1200 rpm. The resulting pellet of human periodontal ligament cells was resuspended in 10 mL Dulbecco’s Modified Eagle Medium (Sigma-Aldrich) supplemented with 10% foetal bovine serum (Gibco, Paisley, UK), amphotericin B (5 μg/mL), streptomycin (0.2 mg/mL), and penicillin (200 U/mL). Cells were cultured in 75-cm^3^ flasks at an initial density of 2.5 × 10^4^ cells/flask. The study was approved by the Ethics Committee of the Pomeranian Medical University in Szczecin (KB-0012/134/18, approval date 26 November 2018) and conducted in accordance with the Declaration of Helsinki. The patients were informed about the study, and written consent was obtained from each participant.

### 2.2. Performing of Periodontal Ligament Cell Cultures and Their Stimulation

Stimulation of periodontal ligament cell cultures was performed according to a previously described procedure [23]. The cells were placed on 24-well plates and incubated overnight. The following day, the cells were washed with phosphate-buffered saline (EURx, Gdansk, Poland), and the medium was replaced with fresh medium containing either no stimulants or the specified agents. Human periodontal ligament cells were stimulated at 0-, 12-, 24-, and 48-h time points with lipopolysaccharide (LPS) (Sigma-Aldrich) at 100 ng/mL and leptin (Peprotech, Thermo Fisher Scientific, Cranbury, NJ, USA) at 10 µg/mL. Leptin and LPS concentrations were selected based on the available literature and verified experimentally by us in preliminary studies [12,23,24,25].

The leptin concentrations used for stimulation of periodontal ligament cells were determined in preliminary studies testing leptin concentrations (0.1, 1, 10, and 50 µg/mL). These concentrations were not toxic to cell cultures, and the cultures survived the entire experiment (up to 48 h) without any significant effect on cell numbers. To stimulate periodontal ligament cells, a leptin dose of 10 µg/mL was selected.

Dulbecco’s Modified Eagle Medium with 10% foetal bovine serum served as the negative control. Each experiment was repeated four times. At each time point, supernatants were collected to determine cytokine concentrations in the culture supernatant using the Luminex assay.

### 2.3. RNA Isolation and RT-qPCR Analysis

Total RNA was isolated and analysed by RT-qPCR according to previously described procedure [23]. Data were normalised using β2-microglobulin (β2M) as the reference gene. Gene expression levels were calculated using the 2^−ΔCt^ method. The primers used for gene expression were presented in Table 1.

### 2.4. Analysis of Cytokine Concentrations in Periodontal Ligament Cell Culture Supernatants

Cytokine concentrations in the supernatants of periodontal ligament cell cultures were quantified using the Luminex Human Discovery Assay (R&D Systems, Minneapolis, MN, USA), in accordance with the manufacturer’s protocol. The panel included IL-1, IL-6, IL-8, IL-10, IL-17, IL-18, and TNF-α.

### 2.5. Statistical Analysis

mRNA expression levels of the cytokines studied, along with the concentrations of their proteins in the culture supernatants of periodontal ligament cells, were compared between study groups using the Kruskal–Wallis test. Where statistically significant differences were observed, the Mann–Whitney test was applied. A value of *p* < 0.05 was considered statistically significant.

## 3. Results

We examined the effect of leptin on the expression of selected cytokines (IL-1, IL-6, IL-8, IL-10, IL-17, IL-18, and TNF-α) in periodontal ligament cells at the mRNA level, as well as the protein levels of these cytokines in culture supernatants. Assessments were carried out after 12, 24, and 48 h of leptin stimulation.

**Figure 1:**
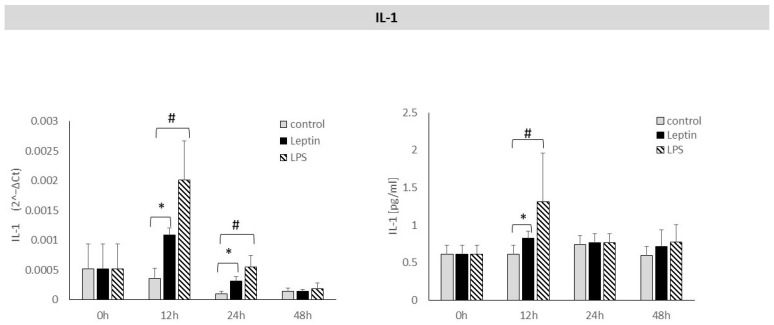
The figure presents mRNA expression and IL-1 protein concentrations in periodontal ligament cell cultures stimulated with leptin or LPS, as a positive control. The mean values ± SD of the results obtained from four replicates are presented. (* *p* < 0.05—cells stimulated with leptin vs. non–stimulated cells), (# *p* < 0.05—cells stimulated with LPS vs. non–stimulated cells).

After 12 and 24 h of stimulation of periodontal ligament cells with leptin, a statistically significant increase in IL-1 mRNA expression was observed. A statistically significant increase in IL-1 protein concentration in culture supernatants was detected after 12 h of leptin stimulation (Figure 1).

**Figure 2:**
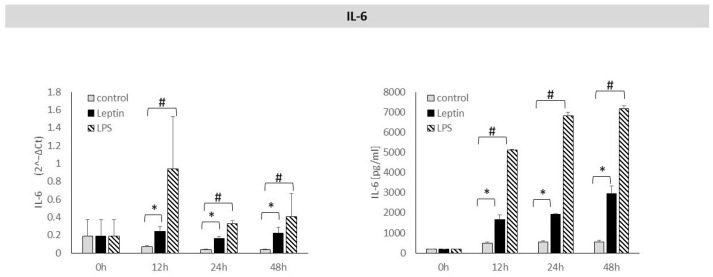
The figure presents mRNA expression and IL-6 protein concentrations in periodontal ligament cell cultures stimulated with leptin or LPS, as a positive control. The mean values ± SD of the results obtained from four replicates are presented. (* *p* < 0.05—cells stimulated with leptin vs. non–stimulated cells), (# *p* < 0.05—cells stimulated with LPS vs. non–stimulated cells).

Leptin stimulation of periodontal ligament cells resulted in a statistically significant increase in *IL-6* mRNA expression after 12, 24, and 48 h. This was accompanied by a statistically significant rise in IL-6 protein levels in the culture supernatants after 12, 24, and 48 h of leptin stimulation (Figure 2).

**Figure 3:**
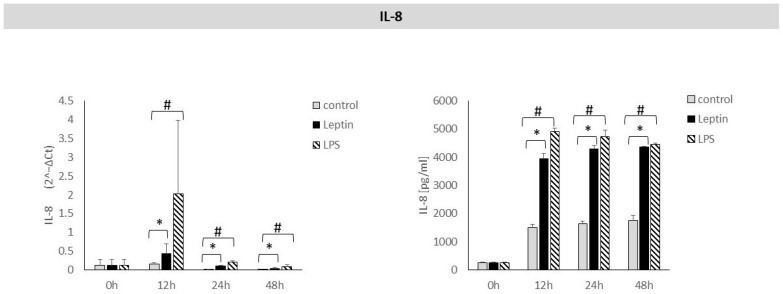
The figure presents mRNA expression and IL-8 protein concentrations in periodontal ligament cell cultures stimulated with leptin or LPS, as a positive control. The mean values ± SD of the results obtained from four replicates are presented. (* *p* < 0.05—cells stimulated with leptin vs. non–stimulated cells), (# *p* < 0.05—cells stimulated with LPS vs. non–stimulated cells).

After 12, 24 and 48 h of stimulation of periodontal ligament cells with leptin, a statistically significant increase in *IL-8* mRNA expression was observed. In the culture supernatants, a statistically significant increase in IL-8 protein concentration was also detected after 12, 24, and 48 h of leptin stimulation (Figure 3).

**Figure 4:**
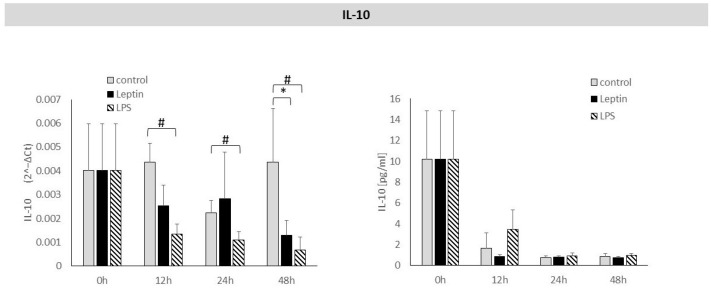
The figure presents mRNA expression and IL-10 protein concentrations in periodontal ligament cell cultures stimulated with leptin or LPS, as a positive control. The mean values ± SD of the results obtained from four replicates are presented. (* *p* < 0.05—cells stimulated with leptin vs. non–stimulated cells), (# *p* < 0.05—cells stimulated with LPS vs. non–stimulated cells).

Leptin stimulation caused a statistically significant decrease in *IL-10* mRNA expression after 48 h. There was no statistically significant effect of leptin on IL-10 protein concentration in culture supernatant after 12, 24, or 48 h of stimulation (Figure 4).

**Figure 5:**
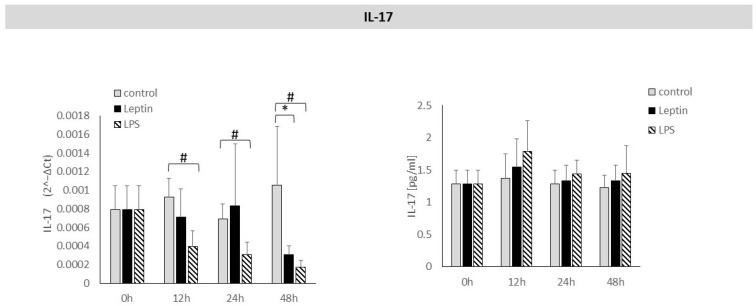
The figure presents mRNA expression and IL-17 protein concentrations in periodontal ligament cell cultures stimulated with leptin or LPS, as a positive control. The mean values ± SD of the results obtained from four replicates are presented. (* *p* < 0.05—cells stimulated with leptin vs. non–stimulated cells), (# *p* < 0.05—cells stimulated with LPS vs. non–stimulated cells).

Leptin stimulation caused a statistically significant decrease in *IL-17* mRNA expression after 48 h. After 12, 24, or 48 h of leptin stimulation, no statistically significant effect of leptin on IL-17 protein concentration in the culture supernatant was observed (Figure 5).

**Figure 6:**
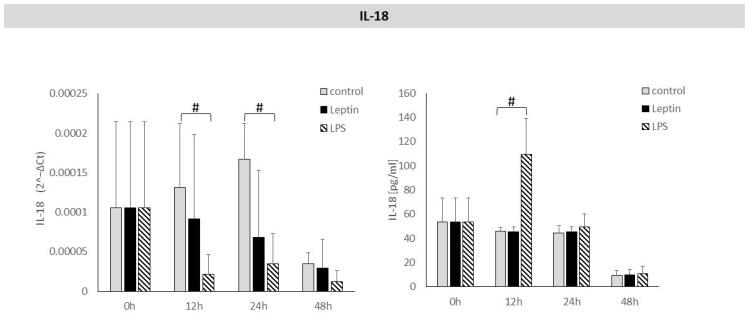
The figure presents mRNA expression and IL-18 protein concentrations in periodontal ligament cell cultures stimulated with leptin or LPS, as a positive control. The mean values ± SD of the results obtained from four replicates are presented. (# *p* < 0.05—cells stimulated with LPS vs. non–stimulated cells).

No statistically significant effect of leptin was observed on *IL-18* mRNA expression in periodontal ligament cells or on IL-18 protein levels in culture supernatants (Figure 6).

**Figure 7:**
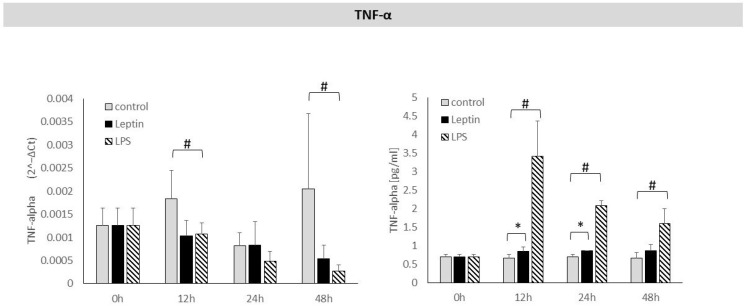
The figure presents mRNA expression and TNF-α protein concentrations in periodontal ligament cell cultures stimulated with leptin or LPS, as a positive control. The mean values ± SD of the results obtained from four replicates are presented. (* *p* < 0.05—cells stimulated with leptin vs. non–stimulated cells), (# *p* < 0.05—cells stimulated with LPS vs. non–stimulated cells).

The effect of leptin on changes in TNF-α expression after 12, 24, and 48 h of stimulation was not observed. However, TNF-α protein levels in the culture supernatants increased significantly after 12 and 24 h of leptin stimulation (Figure 7).

## 4. Discussion

Periodontitis is an inflammatory disease in which numerous cytokines are involved in its pathogenesis. Therefore, in our study, we examined the effect of leptin on cytokine expression in the periodontal ligament cells.

Our results showed a statistically significant effect of leptin on the expression of IL-6 and IL-8 at both the mRNA and protein levels. IL-6 is one of the key cytokines involved in the development of periodontal disease, with elevated levels reported in the blood and gingival fluid of patients with periodontitis. In the case of IL-1, a transient increase in both mRNA expression and protein concentration was observed, persisting up to 24 h of stimulation. With regard to IL-10, a decrease in mRNA expression was noted after 48 h of leptin stimulation, while no significant effect on IL-10 protein concentration was detected in periodontal ligament cell cultures. It is possible that an effect of leptin on IL-10 protein secretion might be observed after stimulation periods longer than 48 h.

There was no statistically significant change in TNF-α mRNA expression after 12, 24, or 48 h of leptin stimulation. The levels of TNF-α protein in the culture supernatant increased after 12 and 24 h of stimulation.

These results can be interpreted in light of previous observations showing that following LPS stimulation, there is a transient increase in *TNF-α* gene expression lasting up to 4 h, after which it declines [26]. Similar results were observed in our previous study, in which we found a statistically significant increase in TNF-α levels in the supernatant of periodontal ligament cell cultures after 12, 24, and 48 h of adiponectin stimulation without a significant effect of adiponectin on TNF-α mRNA expression [25]. The increase in TNF-α levels observed in the present study after 12 and 24 h of leptin stimulation can likely be explained by a transient rise in *TNF-α* gene expression, resulting in the subsequent increase in TNF-α protein synthesis noted at these time points.

Periodontitis is a chronic condition initiated by bacterial infection, which triggers an immune system response. This, in turn, activates immune cells that secrete a range of pro-inflammatory mediators. These mediators interact with various cells and tissues, leading to the production of further mediators that amplify inflammation and contribute to the destruction of periodontal tissues. Pro-inflammatory mediators include numerous cytokines, chemokines, and adipokines. Adipokines are hormones produced by adipose tissue which, in addition to regulating metabolic processes, exert a range of other effects, including both pro-inflammatory and anti-inflammatory actions. Population-based studies have shown a higher prevalence of periodontal disease among obese individuals [6]. Leptin is one of the key adipokines associated with obesity and has been shown to exert pro-inflammatory effects. Nevertheless, the precise role of leptin in the development of periodontitis remains incompletely understood.

The association between leptin and periodontal disease has been demonstrated in both clinical observations and animal model studies. Meta-analyses have confirmed a link between plasma leptin levels and periodontal diseases, with elevated serum leptin levels reported in patients with periodontitis [14,15,16,17,18].

Many studies support a pro-inflammatory role for leptin in the development of periodontitis. However, some evidence suggests that leptin may also exert a protective effect against the disease [19,27]. For example, certain studies have reported decreased leptin levels in the gingival fluid and saliva of patients with periodontitis, along with a negative correlation between gingival fluid leptin levels and disease activity or progression [28]. Most research indicates an increase in plasma leptin concentrations alongside a decrease in gingival fluid leptin levels. This has been explained by the inflammatory process and the associated increase in vascular permeability, which may facilitate the movement of leptin from the gingival fluid into the bloodstream [29,30].

In cellular model studies, leptin has been shown to inhibit M2 macrophages and activate M1 macrophages, which contribute to the development of periodontitis by secreting large amounts of pro-inflammatory cytokines [21]. In addition, leptin has been found to activate the inflammasome of the NOD-like receptor family containing pyrin domain 3 (NLRP3) and to increase M1 polarisation via the NLRP3 inflammasome. Other studies have demonstrated that activated NLRP3 can enhance the synthesis of the pro-inflammatory cytokines IL-1 and IL-18 [31].

A hallmark of periodontitis is the destruction of the alveolar bone. Research has shown that leptin can influence bone metabolism, exerting both destructive and protective effects on alveolar bone. These effects are highly complex and involve multiple signalling pathways and metabolic processes [12].

Leptin affects osteoblasts by reducing the expression of the *c-myc* gene in these cells and increasing the production of cyclin D, which inhibits osteoblast proliferation. In addition, by stimulating protein kinase A, leptin increases the expression of RANKL, leading to osteoclast activation and enhanced bone resorption [32,33]. Leptin may also directly influence periodontal tissue by regulating bone metabolic processes. Furthermore, it can increase bone resorption indirectly by stimulating the synthesis of certain pro-inflammatory factors, such as TNF and IL-6, which are involved in osteoclast activation and the promotion of bone resorption [12,34]. Conversely, some studies indicate that leptin exerts a protective effect on bone tissue. It has been shown to enhance the proliferation of periodontal mucosal cells and the mineralisation of bone-forming cells, thereby promoting osteogenesis. Leptin has also been reported to increase osteoprotegerin synthesis in human gingival fibroblasts, which protects bone by inhibiting osteoclast activation [35]. Evidence further suggests that leptin’s effects on bone metabolism are concentration-dependent [36,37].

In addition to its effects on bone tissue, leptin can also influence other periodontal tissues, exacerbating their destruction—a hallmark of periodontitis. Nokhbehsaim et al. [38] demonstrated that leptin reduces the regenerative capacity of human periodontal ligament cells. Specifically, leptin decreases the expression of growth factors (TGF-β1 and VEGFA), transcription factors (RUNX2), and matrix molecules (collagen and periostin) in human periodontal ligaments. This finding suggests that elevated leptin levels may be one reason for the impaired healing and regeneration of periodontal tissues observed in obese patients.

Schröder et al. [24] investigated the effect of leptin on periodontal ligament fibroblasts under conditions of mechanical loading. Their study showed that leptin increased RANKL expression induced by mechanical loading while reducing osteoprotegerin expression. This suggests that in obese patients, leptin may promote the expression of pro-inflammatory factors and RANKL, while enhancing osteoclastogenesis, thereby accelerating bone resorption.

In our study, we examined the effect of leptin on periodontal ligament cells, which are an important component of the dental ligament and may also be a source of cytokines involved in the development of periodontal disease. We evaluated both the effect of leptin on the expression of selected cytokine genes and on the secretion of cytokine proteins involved in the development of periodontal inflammation. It is crucial to identify cellular responses at both the gene level and the level of secreted protein expression, which is why we chose methods to assess gene expression in cells and protein concentration in the supernatant. The influence of leptin on cytokine gene expression indicates possible modulation of their protein synthesis. However, only the secretion of cytokine proteins into the extracellular space allows cytokines to interact with a number of pathways, including inflammatory processes. Cytokine proteins play a key role in the development of periodontal inflammation. They must be secreted into the extracellular space, where they act on immune response cells such as macrophages and neutrophils, thereby intensifying the inflammatory process. The effect on gene expression alone, without the associated secretion of cytokine proteins into tissues, could be less significant in terms of the severity of inflammation.

The results of our study showed that leptin can influence both the gene expression of some cytokines in periodontal ligament cells and cause the secretion of their proteins into the extracellular space, which may be of significant importance in the development of periodontal inflammation. The results seem to suggest that leptin may be an important stimulator of periodontal inflammation, constituting an significant element in the entire cascade of interactions between cells involved in the development of the inflammatory process. Periodontal inflammation is a very complex process, involving a number of immune system cells and many mediators that stimulate or inhibit each other’s secretion. Our results suggest that leptin, through its effect on the secretion of some cytokine proteins, may be an important element in the processes leading to the development of periodontal inflammation.

Unfortunately, our study has some limitations. We did not examine the effect of leptin on periodontal ligament cells in the presence of LPS, which reflects inflammation. The second limitation is the lack of presentation of leptin stimulation at different concentrations. We hope that despite these limitations, the results of our study will contribute to a better understanding of the role of leptin in periodontal disease.

The results of previous studies indicate that leptin may play a significant role in the development of periodontitis. Our findings suggest that leptin may enhance the expression of cytokines involved in the progression of periodontitis. However, the precise mechanisms by which leptin participates in the pathogenesis of periodontitis remain unclear. Further research is therefore needed to comprehensively determine leptin’s role in the development of periodontal disease.

## 5. Conclusions

Leptin may contribute to the pathogenesis of periodontitis by modulating the expression of certain pro-inflammatory cytokines in periodontal ligament cells.

## Figures and Tables

**Table 1:** Primer sequences used in experiment.

Gene	Forward Sequence	Reverse Sequence
β2-M	5′-AATGCGGCATCTTCAAACCT-3′	5′-TGACTTTGTCACAGCCCAAGA-3′
TNF-α	5′-GATGATCTGACTGCCTGGGC-3′	5′-CACGCTCTTCTGCCTGCTG-3′
IL-1β	5′-ACAGATGAAGTGCTCCTTCCA-3′	5′-GTCGGAGATTCGTAGCTGGAT-3′
IL-6	5′-CACTGGTCTTTTGGAGTTTGAG-3′	5′-GGACTTTTGTACTCATCTGCAC-3′
IL-8	5′-AACCCTCTGCACCCAGTTTTC-3′	5′-ACTGAGAGTGATTGAGAGTGGAC-3′
IL-10	5′-GGTTGCCAAGCCTTGTCTGA-3′	5′-AGGGAGTTCACATGCGCCT-3′
IL-17α	5′-GAGCCCCAAAAGCAAGAGGAA-3′	5′-TGCGGGCATACGGTTTCATC-3′
IL-18	5′-ATCGCTTCCTCTCGCAACAA-3′	5′-CTTCTACTGGTTCAGCAGCCATCT-3′

Abbreviations: β2-M, beta-2 microglobulin; TNF-α, tumour necrosis factor alpha; IL, interleukin.

## Data Availability

Data are contained within the article.

## Abbreviations

The following abbreviations are used in this manuscript:

hPDLC	Human Periodontal Ligament Cells
IL	Interleukin
IFN-γ	Interferon gamma
LPS	Lipopolysaccharides
NF-κB	Nuclear Factor Kappa-Light-Chain-Enhancer of Activated B Cells
PCR	Polymerase Chain Reaction
RA	Rheumatoid Arthritis
RANKL	Receptor Activator for Nuclear Factor κ B Ligand
RNA	Ribonucleic Acid
RT-qPCR	Reverse Transcription Quantitative Polymerase Chain Reaction
TNF-α	Tumor Necrosis Factor Alpha
TGF-β1	Transforming Growth Factor Beta-1
